# Functional and Clinical Characteristics of Cell Adhesion Molecule CADM1 in Cancer

**DOI:** 10.3389/fcell.2021.714298

**Published:** 2021-07-30

**Authors:** Hongxu Li, Jie Gao, Shuijun Zhang

**Affiliations:** ^1^Department of Hepatobiliary and Pancreatic Surgery, The First Affiliated Hospital of Zhengzhou University, Zhengzhou, China; ^2^Key Laboratory of Hepatobiliary and Pancreatic Surgery and Digestive Organ Transplantation of Henan Province, The First Affiliated Hospital of Zhengzhou University, Zhengzhou, China; ^3^Open and Key Laboratory of Hepatobiliary & Pancreatic Surgery and Digestive Organ, Transplantation at Henan Universities, Zhengzhou, China; ^4^Henan Key Laboratory of Digestive Organ Transplantation, Zhengzhou, China

**Keywords:** CADM1, proliferation, metastasis, biomarker, cancer

## Abstract

The cell adhesion molecule CADM1, which participates in cell adhesion and signal transduction, has a regulatory effect on the development of tumors. CADM1 is often involved in malignant tumors of multiple organ systems, such as the respiratory and digestive systems. Upregulated CADM1 promotes tumor cell apoptosis and inhibits malignant proliferation. Along with cell cycle-related proteins, it participates in regulating signaling pathways, such as EMT, STAT3, and AKT, and plays an important role in inhibiting invasion and migration. Considering clinical characteristics, low CADM1 expression is associated with aggressive tumors and poor prognosis. In addition, some long non-coding RNAs (lncRNAs) or miRNAs directly or indirectly act on CADM1 to regulate tumor growth and motility. Interestingly, CADM1 function differs in adult T-cell leukemia/lymphoma (ATLL), and NF-κB is thought to be involved in this process. Taken together, CADM1 could be a potential biomarker for early diagnosis and a target for cancer treatment in future clinical practices.

## Introduction

Cancer has a high mortality rate and is the main factor affecting human health globally (Torre et al., 2016). Clinical manifestations in the early stages of cancer may not be obvious. However, as the disease progresses toward the later stages, clinical manifestations appear prominently usually with a poor prognosis leading to death. While mainstream radiotherapy and chemotherapy are effective, they have several drawbacks. Thus, personalized therapy is expected to become the focus of clinical practice in the future due to accurate diagnosis and better therapeutic effects.

Cell adhesion molecules are proteins located in biological cell membranes, which participate in cell-to-cell recognition, cell activation, and signal transduction during cell motility, proliferation, and apoptosis. They mainly have four groups: integrin, selectin, and cadherin family, and immunoglobulin superfamily (Elangbam et al., 1997).

*CADM1*, also known as *TSLC1*, *IGSF4*, *SgIGSF*, and *SynCAM* (Kuramochi et al., 2001; Watabe et al., 2003), belongs to the immunoglobulin superfamily and was initially identified as a tumor regulator in small cell lung cancer (SCLC). CADM1 protein includes three extracellular immunoglobulin loops, a single transmembrane domain, and a short C-terminal cytoplasmic domain. The cytoplasmic domain contains two conserved protein interaction modules: 4.1 binding motif and a type II PDZ-binding motif (Pujari et al., 2015). The two proteins interact with 4.1 proteins and membrane-associated guanylate kinase homologs (MAGuKs), respectively, and participate in the formation of epithelial cell morphology (Yageta et al., 2002; Sakurai-Yageta et al., 2009) and polarity (Shingai et al., 2003; Wu et al., 2007), along with intracellular signal transduction. Furthermore, CADM1 is located on the lateral side of epithelial cell membranes and mediates adhesion with neighboring cells through trans-homophilic interactions (Koma et al., 2008; Ito et al., 2012). Furthermore, in association with MPP3, DIg, membrane-associated guanylate kinase homolog (MAGuK) proteins, and PI3K, CADM1 forms a multiprotein complex at the periphery of cells. This complex activates the PI3K pathway mediated by CADM1, which results in actin cytoskeleton reorganization and formation of the epithelial cell structure (Murakami et al., 2014). Recently, studies have shown that malignant tumors involving multiple systems lack CADM1 expression, which could be related to epigenetic changes in *CADM1* (Murakami, 2005).

In this article, we have discussed CADM1 expression in various tumors, and its related clinical characteristics. Furthermore, its role in proliferation, invasiveness, and metastasis of malignant tumors has been outlined. CADM1 could be an important biomarker, and it may have an important role in early diagnosis and targeted therapy of malignant tumors.

## Histopathology of CADM1 in Cancer

CADM1 is mainly downregulated in malignant tumors of multiple organ systems due to promoter methylation (Uchino et al., 2003). In particular, the loss of CADM1 expression is correlated with histological grades and cancer prognosis as described below (Table 1).

**TABLE 1:** Summary of the clinical characteristics of CADM1 in malignant tumors.

Cancer type	Cases	Expression	Clinical characteristics	PMID
Ovarian cancer	180	Down	Histological grade, FIGO Stage	33663040
			Lymph node metastasis	
Cervical cancer	131	Down	Histological diagnosis	31067838
Malignant melanoma	95	Down	Overall survival (OS), progression-free survivor	3091107
Nasopharyngeal carcinoma	20	Down	Tumor progression	29070520
Osteosarcoma	92	Down	Overall survival, poor prognosis (PFS)	30013657
Pancreatic cancer	20	Down	Differentiation, lymphatic invasion, TNM stage	28904340
Non-small cell lung cancer	40	Down	Poor overall survival	33325094
Colorectal cancer	54	Down	Dukes’ stage, PT (T1, T2, T3, T4)	20340131
Gastric cancer	120	Down	OS, PFS, TNM stage, differentiation	31389606
Hepatocellular carcinoma	90	Down	T stage, advanced TNM stage, OS	31118799
	82	Down	Recurrence after liver transplantation	21271221
Breast cancer	128	Down	OS, TNM stage, lymph node (LN) metastasis	31579252
			Brain metastasis risk	
Neuroblastoma	28	Down	Disease-free survival, recurrence	27899382
Glioblastoma	30	Down	Median survival, cancer progression	29921422
Bladder cancer	84	Down	Tumor recurrence/size/grade, TNM stage	30719104
Cutaneous squamous cell carcinoma	87	Down	Tumor metastasis, invasive depth	23812766
			Histological grade, OS	
Clear cell renal cell carcinoma	64	Down	AJCC stage, OS	25031695
Gallbladder cancer	37	Down	LN metastasis	24445397
Kaposi’s sarcoma	/	Up	/	29698475
Adult T-cell leukemia/lymphoma	71	Up	Disease progression	31642546
			Prognostic conversion risk	
Small cell lung cancer	28	Up	Pathological stage	33298314
Merkel cell carcinoma	/	Up		33419290
Mycosis fungoides	/	Up		33419291
Sezary syndrome	/	Up		33419292

### Respiratory System

Previous studies frequently evaluate CADM1 expression in malignant tumors of the respiratory system, identify regulatory mechanisms, and determine correlations between CADM1 and clinical prognosis.

Nasopharyngeal carcinoma is a malignant tumor that occurs in and around the nasopharyngeal cavity. CADM1 plays an important role in nasopharyngeal carcinoma (Liu et al., 2017), and its expression is downregulated in nasopharyngeal carcinoma tissues.

Lung cancer is a malignant tumor with high morbidity and mortality (Collins et al., 2007; Hirsch et al., 2017; Oberndorfer and Müllauer, 2018; Romaszko and Doboszyńska, 2018). Small cell lung cancer is an aggressive, high-grade, neuroendocrine tumor. It has been identified that 80% of SCLC cells retain higher CADM1 expression (Funaki et al., 2021). The 4.1 protein binding motif of CADM1 in the cytoplasmic domain promotes colony formation. Furthermore, knockdown of 4.1R inhibits CADM1 mediated colony formation (Funaki et al., 2021). In addition, clinical data suggest that membrane colocalization of CADM1 and 4.1R is associated with higher tumor stage, and CADM1-4.1R complex contributes to SCLC malignancy.

Moreover, CADM1 is downregulated in lung adenocarcinomas as well and is highly correlated with shorter survival (Botling et al., 2013). In primary non-small cell lung cancer (NSCLC) tumors, the CADM1 promoter region is significantly methylated in most cases (44%). Promoter methylation is common in advanced tumors (58%). The level of CADM1 protein is negatively correlated with disease stage, lymphatic involvement, and vascular invasion. The 4-year overall survival of patients varies with CADM1 expression, and patients having high and low CADM1 expression have survival rates of 84 and 7%, respectively (Uchino et al., 2003). In addition, membrane coexpression of CADM1 and LATS2 is highly correlated with the histological type of lung adenocarcinoma (Ito et al., 2019). In particular, low-grade histological subtypes have a higher expression (91 or 100%) than advanced subtypes (41 or 3%). Similarly, membrane coexpression of CADM1 and LATS2 is associated with a better prognosis (5-year disease-free survival rate: 83.8%). Thus, membrane CADM1 detection can be used to diagnose and determine the prognosis of lung adenocarcinoma. It plays an essential role in the histological type and clinical prognosis of tumors, and hence, further research in this direction would be recommended.

### Digestive System Tumors

Hepatocellular carcinoma (HCC) has high morbidity and mortality and is commonly prevalent (Crocetti et al., 2017; Kim et al., 2017; Couri and Pillai, 2019; Luo et al., 2020). Until now, surgical resection and liver transplantation (LT) play an important role in radical treatment. However, due to its invasive nature and recurrence, the 5-year survival rate after surgery is not satisfactory. The poor prognosis of HCC patients after LT is probably related to CADM1 promoter methylation (Zhang et al., 2011). Abnormal CADM1 methylation corresponds to a higher recurrence rate in patients (41.5%) with high CADM1 methylation. Multivariate analysis suggests that the methylation status of CADM1 (Hazard ratio HR = 2.788; 95% confidence interval: 1.043–5.063; *P* = 0.010) is an independent prognostic factor in disease-free survival (DFS) of HCC patients after LT (Zhang et al., 2011).

Epigenetic changes in CADM1 play a critical role in colorectal carcinoma pathogenesis. Chen et al. (2011) identified that CADM1 expression and methylation rate are correlated (*r* = −0.496, *P* < 0.001). *CADM1* methylation results in a decrease in its expression, which promotes the occurrence and development of colorectal cancer. In terms of clinicopathology, CADM1 methylation is highly correlated with T stage and Dukes’ stage of colorectal cancer. Thus, epigenetic changes in CADM1 do play a role in tumor regulation. However, it has not been studied in detail. Therefore, the regulatory network involved in CADM1 expression and promoter methylation should be investigated. This will aid the development and clinical application of demethylation drugs in the future.

Gastric cancer requires new diagnostic markers since it has a low early diagnosis rate (Pasechnikov et al., 2014). CADM1 expression in gastric cancer is low, and its overexpression significantly suppresses tumor metastasis. Honda et al. (2002) identified that the CADM1 promoter is methylated in primary gastric cancer (Pasechnikov et al., 2014).

In addition, CADM1 mRNA and protein expression is associated with laryngeal squamous cell carcinoma cases (Lu et al., 2012). Considering clinical prognosis, lower CADM1 expression is associated with severe laryngeal squamous cell carcinoma (Chang et al., 2016). In esophageal squamous cell carcinoma, the OS (overall survival) and PFS (progression-free survival) of the CADM1 positive group was higher than those of the negative group, and CADM1 could have a potential role in prognosis (Zeng et al., 2016). Moreover, CADM1 inhibits squamous cell carcinoma by reducing STAT3 activity (Vallath et al., 2016). In addition, CADM1 enhances the intestinal barrier function in rats with irritable bowel syndrome with diarrhea (IBS-D) by inhibiting the STAT3 signaling pathway (Sun et al., 2020).

### Nervous System Tumors

Glioblastoma (GBM) is a fatal malignant tumor of the central nervous system (Davis, 2016; Wirsching et al., 2016). CADM1 expression is lower in glioblastoma in comparison to normal brain tissues. Low expression of CADM1 protein and mRNA is evident in the tumor tissues of GBM patients, and the expression level of CADM1 is regulated by exosomal miR-148a (Cai Q. et al., 2018). Furthermore, CADM1 expression is associated with poor median survival and cancer progression in GBM patients, which suggests that CADM1 has an essential role in glioblastoma proliferation and metastasis.

Neuroblastoma is one of the most common extracranial tumors in children (Whittle et al., 2017; Nakagawara et al., 2018). The high expression of CADM1 is significantly associated with higher survival (Seong et al., 2017). Loss of CADM1 in chromosomes (11q23 region) is associated with a poor prognosis in neuroblastoma patients. Clinicopathological analysis suggests that the CADM1 expression level is significantly related to the primary neuroblastoma stage and pathological type according to Shimada classification. Additionally, immunohistochemistry also suggests that low expression of CADM1 correlates with poor prognosis. Thus, CADM1 is associated with nervous system tumors and can act as an effective indicator of tumor progression.

### Reproductive System

Due to the high mortality of gynecological malignancies, it is important to diagnose and treat ovarian cancers early (Kujawa and Lisowska, 2015; Stewart et al., 2019). CADM1 expression in ovarian cancer tissues and cell lines is also significantly low (Si et al., 2020). Furthermore, CADM1 overexpression inhibits metastasis and migration in ovarian cancer cells. Knockdown of CADM1 significantly enhances progression, tumor cell proliferation, and colony formation, while inhibiting apoptosis. In addition, using 180 patients with ovarian serous carcinoma, Wu et al. (2021) found that the correlation between the expression of CADM1 and tumor histological type and lymph node metastasis was statistically significant. Specifically, CADM1 is upregulated in benign serous cystadenomas and serous borderline tumors. On the contrary, it has low expression in malignant tumors, such as low-grade and high-grade serous ovarian cancers. Thus, CADM1 can be a diagnostic indicator and a potential therapeutic target for ovarian cancer.

Cervical cancer is a common gynecological malignancy (Vu et al., 2018; Canfell, 2019). Studies have shown that CADM1 expression is significantly low, and invasion, migration, and angiogenesis of cells in cervical cancer are high. HPV16 E7 infection of cervical cancer cell lines induces CADM1 promoter methylation, which inhibits the expression of CADM1 (Yanatatsaneejit et al., 2020). Moreover, cervical biopsy experiments suggested that the CADM1 promoter methylation rate in cervical cancer is higher than that in normal tissues and low-grade squamous intraepithelial lesion (LSIL) or CIN1 (Bierkens et al., 2013). The severity of cervical lesions is correlated with CADM1 promoter methylation, which suggests that it can be used as a new biomarker in the early diagnosis of cervical cancer.

### Urinary System Tumors

Bladder cancer, which occurs in the bladder mucosa, is the most common malignant tumor of the urinary system (Martinez Rodriguez et al., 2017). In a study considering bladder cancer and normal tissues, CADM1 expression was suppressed by methylation, and its expression was significantly lower in cancer tissues (Chen et al., 2019). CADM1 overexpression regulates caspase-3, Bax, p27, and e-cadherin activity, which promotes cell apoptosis, and reduces invasiveness.

In clear cell renal cell carcinoma as well, the expression of CADM1 is low, and it is involved in regulating cancer cell proliferation, invasion, and migration in association with CADM1-AS1 (Yao et al., 2014).

### Tumors in Other Systems

Breast cancer, with its increasing incidence, is the second highest cause for cancer-related deaths in women (Fahad Ullah, 2019). Breast cancer brain metastasis (BCBM) seriously affects the prognosis of patients and lowers the overall survival (Wikman et al., 2014). In general, primary breast cancer and BCBM patients have low CADM1 expression. Clinically, high CADM1 expression is correlated with disease stage, lymph node status, and tumor size, which results in a higher cumulative survival index (Takahashi et al., 2012; Wikman et al., 2014; Zhang G. et al., 2019). Moreover, CADM1 and DAL-1/4.1B methylation are involved in the invasion, metastasis, and disease progression of primary breast cancer (Takahashi et al., 2012).

Interestingly, CADM1 expression is upregulated in most triple-negative breast cancer cases. However, after primary systemic therapy (PST), patients with incomplete remission have low CADM1 levels, which suggests that CADM1 downregulation is associated with low therapeutic effect of PST in triple-negative breast cancer (Kanke et al., 2019).

Malignant melanoma (MM) is associated with a high mortality rate (Cummins et al., 2006). With early diagnosis and treatment, the melanoma survival rate can be notably improved. CADM1 overexpression inhibits invasion and migration in melanoma (You et al., 2014) and induces the death of tumor cells under non-adhesive conditions. Moreover, low CADM1 expression is significantly associated with poor overall survival or progression-free survival, which suggests that CADM1 could be a prognostic biomarker (Hartsough et al., 2019). Furthermore, immunohistochemistry suggests that CADM1 expression is low, and CADM1 promoter methylation relates to tumor progression and cancer stage. Thus, CADM1 provides new therapeutic targets and prognostic indicators for MM.

In addition, CADM1 acts as a scaffold molecule for immune cells in keratinocytes. CADM1 overexpression promotes permeability and cytotoxicity of T cells and enhances antigen presentation in CD141^+^ dendritic cells. Furthermore, CADM1 promotes vascular endothelial cells to repair the endothelial barrier and mast cells to release cytokines. Interestingly, high CADM1 expression in Merkel cell carcinoma, adult T-cell leukemia/lymphoma (ATLL), mycosis fungoides, and Sezary syndrome is associated with poor prognosis (Magadmi et al., 2019; Sawada et al., 2020). However, this is contrary to previous studies that showed that CADM1 acts as a tumor suppressor in most solid tumors. High CADM1 expression in ATLL cells may be associated with transcriptional abnormalities due to the accumulation of genetic or epigenetic alterations; alternatively, CADM1 may interact directly with the PDZ domain of T-lymphoma invasion and metastasis 1 (Tiam1), promoting lamellipodia formation and activating the Rac-regulated actin cytoskeleton, potentially leading to leukemic cell metastasis in ATLL patients (Nakahata and Morishita, 2012). The specific reasons are unclear, and this is the focus of future related research, which will help us to more fully understand the mechanism of action of CADM1 in hematological tumors compared to solid tumors.

## CADM1 Function

CADM1 functions along with a variety of regulatory molecules and signaling pathways to regulate tumor proliferation, apoptosis, metastasis, and other functions in multiple organ systems (Table 2).

**TABLE 2:** Summary of the function and regulatory factor/pathway for CADM1 in cutaneous malignancies.

Cancer type	Property	Function	Regulatory factor/pathway
Ovarian cancer	Suppressor	Colony formation, apoptosis	PI3K/AKT/mTOR, ROS/JNK, Rap1A, EDN1
		Proliferation, invasion, migration	
Cervical cancer	Suppressor	Invasion, migration, angiogenesis	miR-205, AKT, VEGF, IL-8
		Tumorigenesis	
Malignant melanoma	Suppressor	Invasion, migration, EMT	TWIST1, miR-214, MMP-2/9, Linc01296
		Cell viability, tumorigenesis	RAS, RAF, MEK, ERK1/2
Nasopharyngeal carcinoma	Suppressor	Invasion, proliferation	FRα, ERK1, ERK2
Osteosarcoma	Suppressor	Proliferation, invasion, migration	miR-214-3P, P44/42, MAPK
Pancreatic cancer	Suppressor	Apoptosis, proliferation	miR-196b
Non-small cell lung cancer	Suppressor	Apoptosis, proliferation, invasion	YAP1, MST1/2, LATS1/2, Hippo, miR-423-5p
		Migration, vascular permeability	HER2, STAT3
Colorectal cancer	Suppressor	Tumorigenesis, invasion	c-Src, cbp
Gastric cancer	Suppressor	Proliferation, invasion, migration	LncCAMD1-AS1, miR-126
Hepatocellular carcinoma	Suppressor	Invasion, migration, angiogenesis	PTEN,AKT,GSK-3β,Rb,E2F
		Tumorigenesis	Wint, miR-1246/10b/194/873/155
Breast cancer	Suppressor	Proliferation, invasion, migration	miR-155-3p, DAL-1/4.1B
		Apoptosis	
Neuroblastoma	Suppressor	Invasion, migration	MET-75, SPHK1
Glioblastoma	Suppressor	Invasion, migration, proliferation	LncCAMD1-AS1, miR-148a, STAT3
Prostate cancer	Suppressor	Invasion, migration	ANXA1
Bladder Cancer	Suppressor	Invasion, migration, proliferation	Caspase-3, Bax, EMT
		Apoptosis	
Squamous cell carcinoma	Suppressor	Invasion, migration, proliferation	STAT3, HER2, P14, miR-424-5p
		Apoptosis, EMT	
Clear cell renal cell carcinoma	Suppressor	Migration, apoptosis, cell growth	LncCADM1-AS1
Gallbladder cancer	Suppressor	Migration, proliferation, EMT	miR-10b, TGF-β, miR-182
Kaposi’s sarcoma	Oncogene	Proliferation, tumorigenesis	VFLIP, VGPCR, NF-κB. NEMO
Adult T-cell leukemia/lymphoma	Oncogene	Cell growth, invasion, self-aggregation	HTLE-1, Tax, NF-κB, P47
		Endothelial cell adhesion	
Small cell lung cancer	Oncogene	Proliferation, tumorigenesis	4.1R
Merkel cell carcinoma	Oncogene	Infiltration	/
Mycosis fungoides	Oncogene	Infiltration	/
Sezary syndrome	Oncogene	Infiltration	/

### Proliferation

Malignant tumor progression is often accompanied by abnormal cell proliferation, which affects surrounding tissues, and is regulated by internal molecules and signaling pathways. Here, we have discussed the relationship between CADM1 and cellular proliferation in the context of its function and the molecular mechanism of proliferation.

**FIGURE 1:**
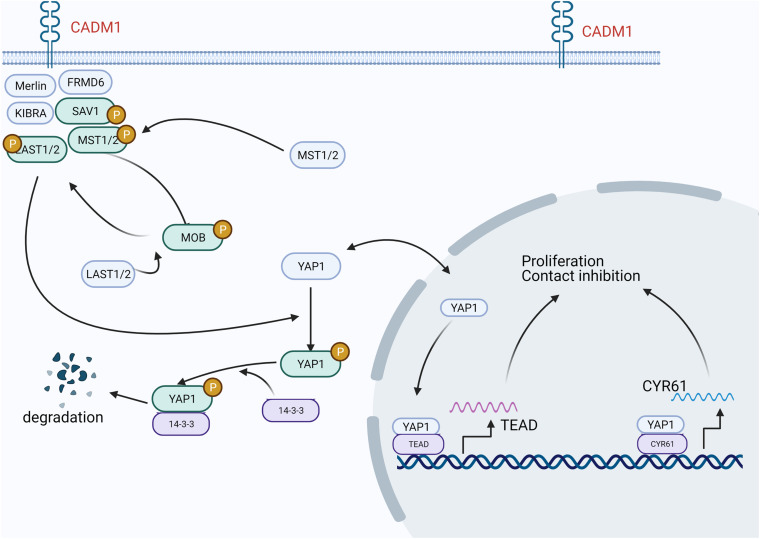
In lung adenocarcinoma, CADM1 participates in the Hippo pathway, thereby regulating cell proliferation and contact inhibition. CADM1 forms a scaffold protein complex with NF2, KIBRA, SAV1, etc., recruits MST1/2 and LATS1/2 kinases to the cell membrane to activate the Hippo pathway, and then moves to the cytoplasm or nucleus after phosphorylation to inactivate YAP1 and inhibit the transcription of its target genes, thereby inhibiting cell proliferation.

In lung adenocarcinoma, CADM1 is involved in the Hippo pathway and regulates cell proliferation and contact inhibition (Figure 1). Specifically, CADM1 forms a scaffold protein complex with NF2, KIBRA, SAV1, etc., recruits MST1/2 and LATS1/2 kinases to the cell membrane, and moves to the cytoplasm or nucleus after phosphorylation to inactivate YAP1. In this manner, activation of the hippo pathway inhibits cell proliferation (Raj and Bam, 2019).

Overexpression of lncCADM1-AS1, which is the antisense transcript of the protein coding gene *CADM1*, can inhibit AKT and GSK-3β phosphorylation by upregulating PTEN (Wang F. et al., 2019). The expression of cell cycle-related proteins cyclin D, cyclin E, CDK2, CDK4, and CDK6 is downregulated, while the expression of p15, p21, and p27 is reversed. Eventually, in the form of p15-cyclinD/CDK4/CDK6 or p21/p27-cyclinE/CDK2, the G0/G1 phase is blocked and the proliferation of HCC cells is inhibited.

Interestingly, in ATLL, CADM1 along with the activated IKK complex promotes NF-κB activation, which, in turn, promotes the proliferation of malignant cells (Sarkar et al., 2019). Similarly, in diseases, such as Kaposi’s sarcoma (KS) and primary exudative lymphoma (PEL) caused by Kaposi’s sarcoma herpes virus/human herpes virus 8 (KSHV/HHV8), CADM1 is possibly involved in chronic NF-κB activation (Hunte et al., 2018).

### Cell Motility

Metastatic progression is complex and includes migration and invasion, vascular intravasation, resistance to anoikis, and extravasation for colonization in a distant organ.

**FIGURE 2:**
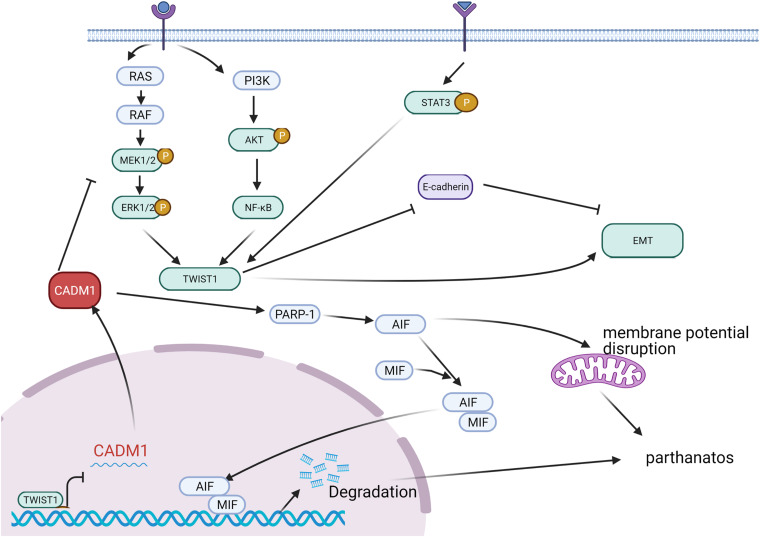
In malignant melanoma, extracellular stimulus signals are transmitted into cells through receptors on the membrane and can activate TWIST1 through RAS-RAF-MEK1/2-ERK1/2, PI3K-AKT-NFKB, and STAT3 pathways, respectively. TWIST1 can inhibit E-cadherin and promote the process of EMT. CADM1, generally highly expressed, can inhibit the activation of TWIST1 by inhibiting the RAS-ERK pathway and then inhibit the EMT process. CADM1 can increase PARP-1 (poly ADP ribose polymerase) and recruit AIF (mitochondrial apoptosis inducing factor) and MIF (microphage migration inhibitory factor) to the nucleus, breaking down DNA into large fragments, and promoting cell death or inducing the destruction of mitochondrial membrane potential, leading to cell-dependent cell death. That is to say, elevated CADM1 can inhibit invasion and migration by inhibiting the EMT process or inducing cell death under non-adhesive conditions.

Epithelial–mesenchymal transition (EMT) plays an essential role in cell invasion and migration. CADM1 actively participates in the invasion and migration of a variety of cancers. Furthermore, CADM1 overexpression inhibits cancer cell metastasis and inhibits cancer progression. In melanoma, TWIST1 directly inhibits CADM1 expression by interacting with the E-box in the CADM1 promoter region and promotes EMT phenotype conversion, which aggravates migration and invasion of melanoma cells (Hartsough et al., 2019). Moreover, CADM1 acts as an inhibitor of the RAS-RAF-MEK1/2-ERK1/2 pathway and affects TWIST1 activation and the EMT process. In this manner, it regulates invasion and migration of melanoma cells (Figure 2). For high-density cells, increased CADM1 expression inhibits TWIST1 activation and inhibits the EMT process. Furthermore, it promotes cell death under non-adhesive conditions to inhibit tumor metastasis through poly ADP ribose polymerase (PARP) (Pascal, 2018; Wang Y. et al., 2019).

In addition, CADM1 binds HER2 through its extracellular domain and activates HER2 as well as CADM1 inhibited, which enhances tumor metastasis by activating the downstream STAT3 signaling pathway. Therefore, the CADM1/HER2/STAT3 axis has broad prospects for the treatment of tumor metastasis (Wu et al., 2021).

### Apoptosis

While acting as a tumor suppressor, CADM1 promotes cell apoptosis by regulating apoptosis-related proteins, caspase, and Bax (Liu et al., 2013), thereby inhibiting the malignant proliferation of tumors. In ovarian cancer, CADM1 overexpression upregulates genes like LXR/RXR, IGF1, IFI44L, and C4BPA, in addition to inhibiting the PI3K/Akt/mTOR pathway as well as affecting the downstream (APP, EDN1, TGFBI, and Rap1A) expression (Si et al., 2020). Under the mediation of the APP and ROS/JNK pathways, the expression of caspase-3 and caspase-8 is increased, which, in turn, promotes cell apoptosis. Similarly, in bladder cancer, upregulated CADM1 promotes cancer cell apoptosis by promoting caspase-3 and Bax protein expression, thereby inhibiting tumor growth (Chen et al., 2019).

### Tumorigenesis

The extracellular domain of CADM1 has high homology with other immunoglobulin superfamily CAMs and functionally mediates the formation of CADM1’s own homodimer or heterodimer with other members of CAMs. In this way, cell adhesion is enhanced and invasion and cancer morphogenesis suppressed (Hirohashi and Kanai, 2003).

CADM1 relies on its cytoplasmic domain to play an inhibitory role in tumorigenesis (Mao et al., 2003). CADM1 can achieve tumor suppression through 4.1 binding motif and 4.1B/DAL1 binding, or participate in cbp-dependent c-Src regulation, thereby inhibiting tumorigenicity in nude mice.

C- -Src is a non-receptor tyrosine kinase that is highly activated in colon cancer and induces the malignant transformation of cancer cells (Tsuboi et al., 2020). Mechanistically, the activation of c-Src is regulated by the phosphorylation of tyrosine residues Tyr418 (Y418) and Tyr529 (Y529) (Place et al., 2011). Phosphorylation of the Y418 domain promotes c-Src activation. In contrast, phosphorylation of the C-terminal tail of Y529, catalyzed by Src kinase (Csk), leads to c-Src inactivation. Csk binding protein (Cbp) is a transmembrane junction protein that is in lipid rafts and recruits Csk to the cell membrane. As Cbp recruits Csk and activates c-Src to lipid rafts, Csk phosphorylates c-Src Y529, thereby inactivating the catalytic activity of c-Src. In summary, the CADM1-cbp-c-Src complex is involved in tumorigenesis.

## CADM1 as a Biomarker for Cancer Diagnosis and Prognosis

Based on the clinicopathological manifestations described above, CADM1 can be utilized as a more practical clinical biomarker (Lee et al., 2013; Chen et al., 2016). The specific reason is that CADM1 is downregulated in most cases due to its promoter methylation as well as being related to the progression of malignant tumors, advanced classification, poor 5-year survival, and high recurrence rate; for example, in cervical cancer, CADM1 can be used as a biomarker to participate in the assessment of cervical epithelial lesions (Del Pino et al., 2019; El Aliani et al., 2021). A low level of CADM1 is often associated with more severe cervical lesions.

The NF-κB signaling pathway is thought to be involved in ATLL, and the overexpression of CADM1 in ATLL cells has been identified as a surface marker for human T-cell leukemia virus (HTLV-1) infection of T cells (Nakahata and Morishita, 2012; Nakahata et al., 2012; Pujari et al., 2015; Makiyama et al., 2019). CADM1 promoted the self-aggregation of ATLL cells, adhesion to endothelial cells, and increased tumor growth and invasion in xenograft mice. Based on clinical and experimental data from 71 cases, Junya Makiyama found that people infected with HTLV-1 can be divided into four stages according to the percentage of CD4^+^ CADM1^+^ cells, such as G1 (CADM1 + ≤10%), G2 (10% < CADM1 + ≤25%), G3 (25% < CADM1 + ≤50%), and G4 (50% < CADM1+). The conditions of G1 and G2 are more stable, the risk of G3 is increased, while 28.4% of G4 patients receive systemic chemotherapy for 3 years (Makiyama et al., 2019). This result not only corresponds to the previous expression of CADM1 in ATLL but also demonstrates the feasibility of using CADM1 as an indicator to predict clinical prognosis. In addition, CADM1 can be used as a biomarker for the differential diagnosis of osteosarcoma and chondrosarcoma (Inoue et al., 2013). In view of these findings, we believe that CADM1 as a biomarker will play an increasingly important role in tumor diagnosis and prognosis monitoring.

## Possible Targeted Therapy and Clinical Application of CADM1

In melanoma, TWIST1 can directly act on the CADM1 promoter region to inhibit the expression of CADM1 and promote tumor metastasis. Some common oncogenes are negatively regulated by CADM1 but have a synergistic relationship with the tumor suppressor gene Rb (Zhang et al., 2016). Anti-CADM1 antibody, with its antibody-dependent cell-mediated cytotoxicity, plays an increasingly important role in suppressing the interaction between endothelial cells and CADM1-positive ATLL cells (Chilmi et al., 2020). In addition, core enzymes, such as the Hippo pathway are associated with CADM1. The antitumor actions of CADM1 mediated by dual-regulated oncolytic adenovirus have been demonstrated in a mouse model (Lei et al., 2013). These findings provide new research directions.

The low expression of CADM1 in malignant tumors of multiple systems often involves the regulation of lncRNAs and miRNA (Qin et al., 2014; Mou et al., 2019; Shi et al., 2019; Wang F. et al., 2019). In HCC, the lncRNA DLX6-AS1 is involved in the STAT3 signaling pathway, which regulates the expression of CADM1 and participates in tumor progression (Wu et al., 2019). High expression of CADM1 promotes the apoptosis of pancreatic cancer cells, while miR-196b inhibits apoptosis and promotes the proliferation of pancreatic cancer by targeting the 3′-UTR region of CADM1 (Wang et al., 2017). In addition, immunohistochemistry showed that 40 of the 49 cases of osteosarcoma (>80%) showed low expression of CADM1. miR-214-3p, targeting the CADM1 3′-UTR, inhibits CADM1 and activates the P44/42 mitogen-activated kinase (MAPK) signaling pathway, thereby promoting the invasion, migration, and proliferation of osteosarcoma. Many studies have shown that CADM1 interacts with a single non-coding RNA, such as microRNAs and lncRNAs, thereby participating in the regulation of the occurrence and development of malignant tumors. Whether there is an interaction network between CADM1 and a variety of non-coding RNAs, related genes, and signal pathways and its specific regulatory routine needs to be further verified in the future.

**FIGURE 3:**
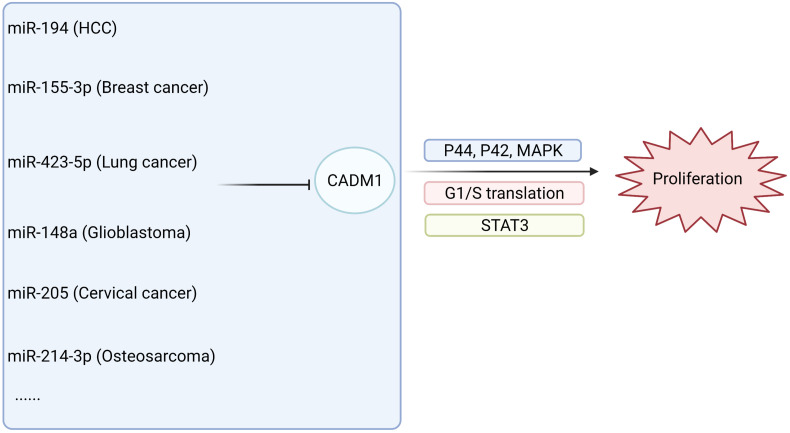
MiRNAs are involved in the proliferation of malignant tumors by acting on CADM1 in multiple systems.

**FIGURE 4:**
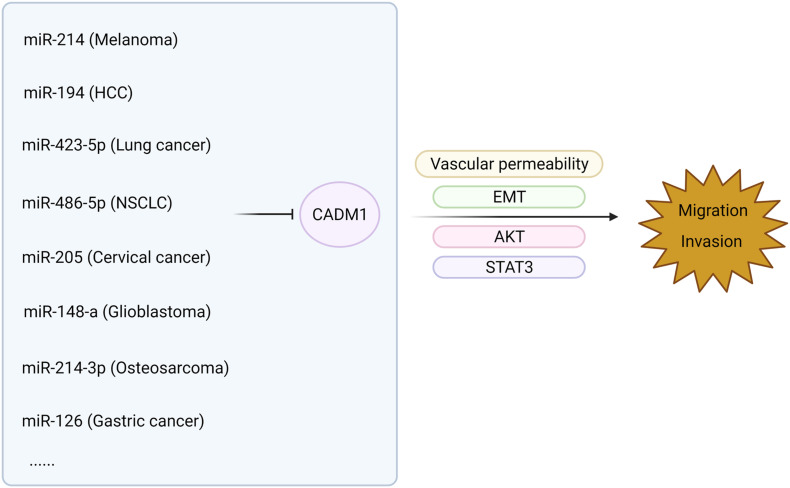
MiRNAs are involved in the metastasis of malignant tumors by acting on CADM1 in multiple systems.

The relationship between CADM1 and related miRNAs through corresponding pathways to regulate tumor proliferation and invasion of multiple systems is shown in Figures 3, 4 (Li et al., 2012, 2019; Qiu et al., 2014; Sun et al., 2014, 2021; Yang et al., 2015; Cai H. et al., 2018; Cai Q. et al., 2018; Han et al., 2018; Zhang F. et al., 2019; Zhang G. et al., 2019; Niu et al., 2020; Wang et al., 2020; Huang and Feng, 2021). Among them, miR-1246 inhibits CADM1 expression by acting directly on the 3′UTR of CADM1, thereby promoting invasive migration of HCC cells in an *in vitro* assay. *In vitro* studies also demonstrated that miR-205 targets CADM1 and that downregulated miR-205 inhibits cervical cancer cell invasion and angiogenesis through the CADM1-mediated Akt signaling pathway, as validated by *in vivo* experiments in nude mice. In clinically targeted therapies, a drug that inhibits DNA hypermethylation, OD-2100, has shown efficacy in anti-ATLL activity and inhibition of tumor growth. In addition, the anti-CADM1 antibody inhibits the interaction of endothelial cells with CADM1^+^ ATLL cells, thereby suppressing tumor metastasis. In short, targeting research involving CADM1 gene, CADM1 molecules on cell membranes, or soluble CADM1 in serum will drive further progress in CADM1-targeted therapy.

## Conclusion

Here, we have summarized the role of CADM1 in multisystem malignancies, discussed the limitations of previous studies, and suggested possible future research directions. Based on the studies discussed herein, CADM1 is involved in the regulation of tumor formation by participating in the EMT, Hippo, AKT, MAPK, and other related signaling pathways. In multiple malignant tumors (HCC, ovarian cancer, lung cancer, neuroblastoma bladder cancer, etc.), high CADM1 expression inhibits malignant proliferation, invasion, and metastasis and promotes tumor cell apoptosis. Low CADM1 expression is often associated with higher clinicopathological types and poor clinical prognosis. However, in individual tumors, such as ATLL and SCLC, CADM1 promotes tumor progression. In addition, non-coding sequences, such as lncRNAs and miRNAs, participate in regulating tumor progression along with CADM1. Therefore, for the diagnosis and targeted therapy of multiple malignant tumors, CADM1 would be a promising biomarker.

## Author Contributions

JG helped to draft the manuscript. HL conceived of this review manuscript and drafted the manuscript. SZ conceived of the work and participated in the design and coordination of this work. All authors collaborated to carry out the presented work, and read and approved the final manuscript.

## Conflict of Interest

The authors declare that the research was conducted in the absence of any commercial or financial relationships that could be construed as a potential conflict of interest.

## Publisher’s Note

All claims expressed in this article are solely those of the authors and do not necessarily represent those of their affiliated organizations, or those of the publisher, the editors and the reviewers. Any product that may be evaluated in this article, or claim that may be made by its manufacturer, is not guaranteed or endorsed by the publisher.

## Abbreviations

lncRNAs: long non-coding RNAs
ncRNAs: non-coding RNAs
miRNAs: microRNAs
mRNA: messenger RNA
EMT: epithelial-mesenchymal transition
HCC: hepatocellular carcinoma
STAT3: signal transducer and activator of transcription (STAT) 3.
